# Colorectal Cancer Study of Austria (CORSA): A Population-Based Multicenter Study

**DOI:** 10.3390/biology10080722

**Published:** 2021-07-28

**Authors:** Andrea Gsur, Andreas Baierl, Stefanie Brezina

**Affiliations:** 1 Institute of Cancer Research, Department of Medicine I, Medical University of Vienna, 1090 Vienna, Austria; stefanie.brezina@meduniwien.ac.at; 2 Department of Statistics and Operations Research, University of Vienna, 1010 Vienna, Austria; andreas.baierl@univie.ac.at

**Keywords:** colorectal cancer, biomarker, genomics, proteomics, metagenomics, screening, biobank

## Abstract

**Simple Summary:**

The Colorectal cancer Study of Austria (CORSA), an ongoing multicenter prospective case‒control study, was initiated to discover prognostic as well as diagnostic biomarkers for colorectal cancer (CRC) risk prediction mainly based on OMICS research, such as genomics, metabolomics, and metagenomics centered at the Institute of Cancer Research at the Medical University of Vienna; recruitment for CORSA started in 2003. Until now, we have generated genomics data, untargeted and targeted metabolomics data, folate-dependent one-carbon metabolism data, and leukocyte telomere length data using the CORSA biobank. The generated data, the collection of biological samples (genomic DNA, plasma, fecal samples) and the comprehensive CORSA database represents a valuable resource for ongoing and future national and international cooperation projects on CRC research.

**Abstract:**

The Colorectal cancer Study of Austria (CORSA) is comprised more than 13,500 newly diagnosed colorectal cancer (CRC) patients, patients with high- and low-risk adenomas as well as population-based controls. The recruitment for the CORSA biobank is performed in close cooperation with the invited two-stage CRC screening project “Burgenland PREvention trial of colorectal Disease with ImmunologiCal Testing” (B-PREDICT). Annually, more than 150,000 inhabitants of the Austrian federal state Burgenland aged between 40 and 80 are invited to participate using FIT-tests as an initial screening. FIT-positive tested participants are offered a diagnostic colonoscopy and are asked to take part in CORSA, sign a written informed consent, complete questionnaires concerning dietary and lifestyle habits and provide an ethylenediaminetetraacetic acid (EDTA) blood sample as well as a stool sample. Additional CRC cases have been recruited at four hospitals in Vienna and a hospital in lower Austria. A major strength of CORSA is the population-based controls who are FIT-positive and colonoscopy-confirmed to be free of polyps and/or CRC.

## 1. Introduction

Colorectal cancer (CRC) is the fourth most common cancer and the third highest cause of cancer-related deaths in Europe, thereby representing a severe public health problem [1]. In Austria, the CRC incidence rate is observed in the lower third within the European Union with about 49.2 per 100,000 inhabitants (Statistics Austria, 2017). CRC is a complex disease with both genetic and lifestyle factors contributing to individual risk of CRC [2]. Early detection of CRC is an important issue since the stage at diagnosis remains the most important prognostic factor for CRC. The 5-year survival rate is about 90% for those patients diagnosed at an early stage but decreases to 10% for advanced or metastasized cancer [3]. The majority of sporadic CRCs develop from normal epithelium through sequentially worsening degrees of adenomatous dysplasia, known as the adenoma-carcinoma sequence [4]. Due to the slow progression of CRC, there is a window of opportunity of about ten years for prevention and intervention.

Screening programs have the potential to detect early precancerous lesions and perform endoscopic removal of adenomas, thereby contributing to the reduction of CRC incidence and mortality [5]. Therefore, population-wide screening programs are recommended in several European countries. Many CRC screening programs utilize a two-stage process wherein patients testing positive on a fecal occult test are followed up by colonoscopy. Nowadays, the preferred approach in testing for occult blood in feces used for CRC screening programs is the fecal immunochemical test (FIT), despite its relatively low specificity and sensitivity. Moreover, FIT tests can detect only a small percentage of non-advanced colorectal adenomas [6].

In Austria, the organization of screening programs is not regulated nationwide; it is a matter of the federal states. To date, only two invited screening programs are performed in Austria, in the federal states Vorarlberg and Burgenland. In Vorarlberg, a colonoscopy-based screening program is offered to the insured population older than 50 years since 2007. In Burgenland, the screening program “Burgenland Prevention Trial of Colorectal Cancer Disease with Immunological Testing” (BPREDICT), initiated in 2003, is a two-stage invited screening program for individuals aged between 40 and 80 using FIT as the initial screening. Participants with a positive FIT were invited for further diagnostic work-up such as a diagnostic colonoscopy. These participants were asked to take part in the “Colorectal Cancer Study of Austria” (CORSA), sign a written informed consent, complete questionnaires, and provide an ethylenediaminetetraacetic acid (EDTA) blood sample as well as a stool sample for the CORSA biobank. In cooperation with B-PREDICT, several hospitals in Vienna and the Hospital Wiener Neustadt, we have established the CORSA biobank, which currently comprises more than 13,500 participants. From each participant, genomic DNA and plasma samples are stored at −80 °C. Two years ago, we also started recruitment of stool samples within the CRC screening program B-PREDICT.

The recruitment of participants for the ongoing CORSA started over 18 years ago at the Medical University of Vienna, Austria. The initial motivation of CORSA was to identify genetic risk variants that modify individual predisposition to CRC. At the beginning, the aim was the identification of single nucleotide polymorphisms (SNPs) as well as tandem repeat minisatellites and their association with CRC risk in a case‒control study design [7]. With the progress in molecular biologically methods, we have focused on high-throughput OMICS-based projects, in particular, genomics, metabolomics and metagenomics. We conducted a genome-wide association study (GWAS) for sporadic CRC and untargeted as well as targeted metabolomics profiling. The most recent CORSA project aims at the identification of diagnostic signatures along the colorectal adenoma-carcinoma sequence analyzing fecal samples via gut metagenomics. Altogether, CORSA aims to discover and validate prognostic as well as diagnostic biomarkers for CRC risk prediction nowadays based mainly on OMICS research.

The aim of CORSA is to identify new robust diagnostic biomarkers for advanced adenomas and early-stage CRC by using state-of-the-art OMICS technologies such as metabolomics, proteomics, and metagenomics in order to improve the identification of patients at higher CRC risk.

## 2. Materials and Methods

### 2.1. Participant Recruitment

CORSA is an ongoing population case‒control study of women and men recruiting newly diagnosed CRC patients (colon, rectum or rectosigmoid cancer (ICD-10 C18-20), histopathologically confirmed invasive cancer of any stage), high-/low-risk adenomas and population-based colonoscopy-negative controls, with an age range between 30 and 90 years. Blood and fecal samples, dietary/lifestyle questionnaires, and anthropometrics/demographics were obtained from each participant.

**Figure 1:**
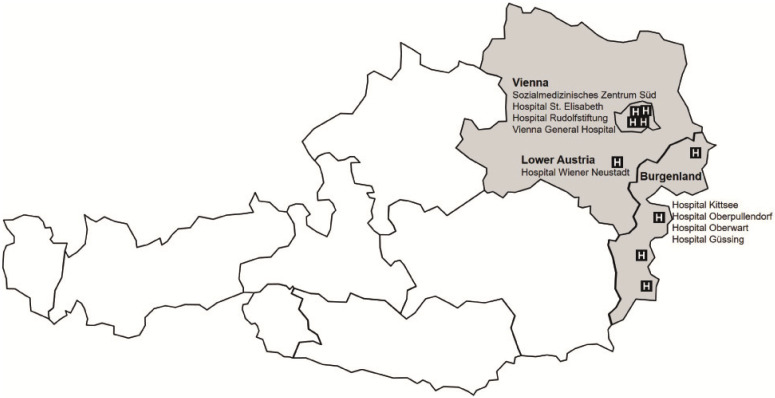
Study sites of CORSA recruitment in Vienna, Burgenland, and lower Austria. Since 2003, more than 13,500 participants have been recruited across nine sites in Austria. Source of illustration: CORSA study team, 2020.

Since 2003, more than 13,500 participants have been recruited across nine sites in Austria. The multicenter recruitment within CORSA follows standardized protocols, resulting in consistent data from all recruitment sites. These sites include the Medical University of Vienna (Department of Surgery), three further hospitals in Vienna (Sozialmedizinisches Zentrum Süd, Hospital Rudolfstiftung and Hospital Elisabeth), and the Hospital Wiener Neustadt in Lower Austria. Furthermore, the recruitment for CORSA was performed in four hospitals in Burgenland (Hospital Kittsee, Hospital Oberpullendof, Hospital Oberwart, Hospital Güssing) within the population-based screening program “Burgenland PREvention Trial of colorectal cancer DIsease with ImmunologiCal Testing” (B-PREDICT) (Figure 1).

B-PREDICT is an invited two-stage screening project initiated by gastroenterologist Karl Mach at the Hospital Oberpullendorf, Burgenland. Annually, more than 150,000 inhabitants of Burgenland aged between 40 and 80 are invited to participate in this program using a fecal immunochemical test (FIT) as an initial screening. Nowadays, FIT is the preferred approach in testing for occult blood in feces used for CRC screening programs [6]. FIT-positive (≥10 µg hemoglobin/g feces) tested individuals are offered a complete colonoscopy and are asked to participate in CORSA and provide an EDTA blood sample, questionnaire, and written informed consent for the CORSA biobank. Within B-PREDICT, we have recruited CRC patients, patients with high- and low-risk adenomas as well as population-based controls. These controls are triggered by a positive FIT result, and because all of them underwent a colonoscopy, they were known to be free of polyps and CRC. The high-risk adenoma group included patients with adenomatous tubular polyps >1 cm, adenomatous tubulo-villous polyps, adenomatous villous polyps, sessile serrated polyps (SSA) and traditional serrated polyps (TSA). Adenomatous tubular polyps <1 cm were considered as low-risk polyps. Baseline characteristics of recruited CORSA participants are given in Table 1.

FIT-positive participants from the B-PREDICT screening are recruited when undergoing colonoscopy and comprise CRC patients, high- and low-risk polyps and colonoscopy-negative controls. CORSA participants from other centers are recruited before surgery or at the time of their clinical follow-up and are mainly CRC cases. All subjects gave written informed consent, and the study was approved by the institutional review boards.

### 2.2. Biospecimen Collection

Genomic DNA, plasma (divided into aliquots in barcoded tubes) and stool samples are collected at each CORSA site using harmonized protocols and stored at −80 °C. Paraffin-embedded (FFPE) tissue from CRC cases is stored at room temperature.

Within CORSA, no regular sequential follow-up recruitment is performed.

Blood draws are performed at least at baseline (first participation in CORSA). In addition, 2263 CORSA participants (17%) have been repeatedly recruited. On average, these participants were recruited about 2.3 times. The mean time period between single participations amounts to 2.9 years. Some participants have a follow-up of about 15 years.

Survival data, representing a comprehensive output file comprising confirmed or unconfirmed date of death, date of last contact, and information on missing or incorrect data input, are provided through a biennial clinical data abstraction from the IT database of the Medical University of Vienna, “Allgemeines Krankenhaus Information Management” (AKIM) in cooperation with Statistics Austria. In addition, we abstract survival data in cooperation with the Main Association of Austrian Social Insurance Institutions (“Hauptverband der österreichischen Sozialversicherungsträger”). Therefore, the social insurance number, first and last name, date of birth, and sex of all participants were processed through a database pipeline. For a subset of the data, the cause of death in terms of ICD-10 category codes of the World Health Organization can be obtained.

### 2.3. Clinical Data

Clinical data were abstracted from medical records and processed in a structured database following standardized documentation guidelines and according to the General Data Protection Regulation (GDPR). The CORSA databank comprises structured information on diagnosis, treatment, histology, progression (recurrence and metastasis), and survival data (Table 2). Follow-up on clinical data is regularly performed. Some CORSA participants have been followed up for about 15 years.

### 2.4. Questionnaires

CORSA participants provided at least a basic CORSA questionnaire assessing data on body mass index (BMI), smoking history, alcohol consumption, education level, family status, profession, basic dietary habits, and diabetes (Table 2). Patient information and interviewing was performed by trained study personnel.

Additionally, CORSA participants were asked to complete a slightly modified version of the VITamins And Lifestyle cohort study (VITAL) questionnaire and the European Prospective Investigation into Cancer and Nutrition Food Frequency Questionnaire (EPIC-Potsdam FFQ2). The VITAL questionnaire assesses the use of nutritional supplements, vitamins, medication, physical activity, and social environment [8]. The EPIC-Potsdam FFQ2 questionnaire was designed to capture detailed dietary habits and is used to receive habitual dietary food or nutrient intake [9].

## 3. Result

### 3.1. Generated Data

So far, we have generated genomics data, metabolomics data, folate-dependent one-carbon metabolism data, and leukocyte telomere length data from the CORSA biobank (Table 3). GWAS data are available from 2677 CORSA participants using the Axiom Array Human Genome-Wide CEU1 array (Affymetrix, Santa Clara, CA, USA) comprising 1060 CRC, 689 high-risk polyps, and 928 colonoscopy-negative controls. Metabolomics data, targeted as well as untargeted, are available from 88 CRC, 200 high-risk polyps, 200 low-risk polyps, and 400 controls. Untargeted metabolomics was conducted using ultrahigh-performance liquid chromatography quadrupole time-of-flight mass spectrometry (UHPLC-qTOF-MS, Agilent Technologies, Santa Clara, CA, USA). Samples for targeted metabolomics were analyzed with the AbsoluteIDQTM p180 kit (Biocrates Life Sciences, Innsbruck, Austria). Both analyses were performed at the biomarker lab headed by Augustin Scalbert (International Agency for Research on Cancer, Lyon, France).

Furthermore, data on folate and biomarkers related to one-carbon metabolism are available from 245 CRC patients at three follow-up time points: baseline (*n* = 218), 6 months (*n* = 10), and 12 months (*n* = 17). The analytical measurements were performed at Bevital AS, Bergen, Norway. Conducting liquid chromatography tandem mass spectrometry (LC-MS-MS) circulating folate, folate derivatives, and folic acid were detected. Gas chromatography tandem mass spectrometry (GC-MS-MS) was performed to analyze other amino acids. C-reactive protein, cystatin C, and its variants were measured by matrix-assisted laser desorption/ionization-time of flight (MALDI-TOF) mass spectrometric analysis.

Telomere length data from 2011 CORSA participants (384 CRC, 544 high-risk polyps, 537 low-risk polyps and 546 controls) were determined using monochrome multiplex quantitative PCR (MMQPCR).

### 3.2. Key Findings and Publications

CORSA key publications are based on genomics and metabolomics: “Bayesian and frequentist analysis of an Austrian genome-wide association study of colorectal cancer and advanced adenomas”, “Plasma metabolites associated with colorectal cancer: a discovery replication study” and “Plasma metabolites associated with colorectal cancer stage: findings from an international consortium” [10,11,12]. All publications related to CORSA are listed in Supplementary Table S1 [13,14,15,16,17,18,19,20,21,22].

We have performed a GWAS of CORSA participants comprising 1060 CRC patients, 689 patients with advanced colorectal adenomas, 928 colonoscopy-negative controls, and additionally, 3439 controls from the “Cooperative Health Research in the Region of Augsburg” (KORA) using Axiom Arrays CEU 1 (Affymetrix, Santa Clara, CA, USA). We pursued a dual approach to investigate genome-wide associations with disease risk applying both single marker analysis as well as model selection based on the modified Bayesian information criterion, mBIC2, implemented in the software package MOSGWA. The advantage of the model selection approach is its larger power to detect candidate SNPs compared to single marker tests. Furthermore, 56 SNPs that are already known to influence CRC susceptibility from previous studies were tested in a hypothesis-driven approach and some of them were also found to be relevant in CORSA. Furthermore, we found some so-far unreported SNPs [10].

This GWAS was the basis for many international cooperation projects: the Genetics and Epidemiology of Colorectal Cancer Consortium (GECCO) at the Fred Hutchinson Cancer Center, Seattle, Washington, two European Cooperation in Science and Technology (COST) Actions “Cooperation studies on inherited susceptibility to colorectal cancer” (BMI1206) and “Identifying biomarkers through translation research and prevention and stratification of colorectal cancer” (CA17118).

Within the “Metabolomic profiles throughout the continuum of colorectal carcinogenesis” (MetaboCCC) consortium, we performed an untargeted metabolomics study comprising 268 CRC patients and 353 controls using independent discovery and replication sets from two European cohorts, the ColoCare Study from Germany and CORSA. The aim of this study was to identify circulating plasma metabolites associated with CRC and to improve knowledge regarding CRC etiology. Multiple logistic regression models were used to test the association between disease state and metabolic features. We identified 691 statistically significant features in the discovery cohort. Testing the second cohort narrowed it to 97. These corresponded to 28 metabolites, of which 15 could be identified [11].

Furthermore, we performed a targeted metabolomics profiling within the MetaboCCC consortium aiming to identify biomarkers related to CRC progression. We investigated plasma concentrations of 130 metabolites from 744 CRC patients (stages I-IV) from two Dutch cohorts (COLON, EnCoRe), the ColoCare study from Germany, and CORSA. Our results suggest that metabolic pathway involving citrulline, histidine, and other molecules that have been previously implicated in CRC development may also be linked to progression [12].

## 4. Discussion

CORSA has multiple strengths bringing together a multidisciplinary team of molecular biologists, clinicians, laboratory scientists, and statisticians to foster research on CRC. The close collaboration with clinicians and clinical centers allows a regular and comprehensive clinical follow-up as well as the automated abstraction of data such as survival information. The multicenter cohort standardized the recruitment of patients, with uniform protocols for the collection, processing, and management of biospecimens. Participants were mainly Caucasian and geographically and ethnically uniform. The comprehensive biological samples and harmonized data provides the basis for national and international cooperation projects on CRC research. Therefore, CORSA represents an active and leading player in cooperation, support actions, and consortia. So far, CORSA contributed to multiple international consortia such as Genetics and Epidemiology of Colorectal Cancer Consortium (GECCO) [23,24,25,26,27,28,29,30,31,32,33,34,35,36,37,38,39,40,41,42,43,44,45,46,47,48,49], COlorectal cancer GENeTics (COGENT) and COnsortium of METabolomics Studies (COMETS) [13]. Furthermore, CORSA was a partner in two ERANET-TRANSCAN-funded projects: MetaboCCC [11,12,44,45,46,50,51], and “Biomarkers related to folate-dependent metabolism in colorectal cancer recurrence and survival” (FOCUS) [35,36]. These interdisciplinary projects bring together multiple European CRC cohorts, including CORSA, the ColoCare study of the German Cancer Research Center in Heidelberg [14], the EnCoRe study of Maastricht University, the Netherlands [15], as well as the COLON study of Wageningen University, the Netherlands [16]. In the course of this collaboration study, data have been pooled and harmonized to increase the statistical power for joint analysis.

A major strength of the biobank is the colonoscopy-negative control group, known to be free of polyps and CRC because all participants underwent a complete colonoscopy within the screening project B-PREDICT.

Nevertheless, some limitations have to be mentioned. CORSA high-risk and low-risk adenomas as well as controls are mainly recruited within the screening program B-PREDICT from centers in Burgenland rather than from centers in Vienna. The reason is that in Vienna no organized CRC screening program has been performed so far; therefore, no colonoscopy-negative controls can be recruited. In Austria, the organization of CRC screening programs is not regulated nationwide; it is a matter of the federal states. Within CORSA, no regularly invited, targeted recruitment at defined follow-up time points is performed.

A new research focus of CORSA besides genomics and metabolomics is the gut microbiome. Recently, the project “Gut MICRObiome-based approach for incorporating new biomarkers into COLOrectal cancer screening” (MICROCOLO) was funded by the Austrian Research Promotion Agency (FFG). There is evidence that changes in the gut microbiome occur during different stages of colorectal neoplasia, supporting an etiologic and diagnostic role for the microbiome. Harnessing knowledge of the microbiota may lead to new preventive strategies and diagnostics. myBioma GmbH, our company partner within this FFG-funded project, will use their already established data processing and statistical analysis workflow to process the resulting microbiome data. Predictive microbiome-based signatures specific for CRC and high-risk adenomas will be defined by multivariate statistical modelling. The combination of conventional screening methods such as FIT with microbiome-based methods is a promising tool for early detection of CRC and could improve diagnostic accuracy.

## 5. Conclusions

The CORSA biobank, comprising genomic DNA, plasma, fecal samples, and a comprehensive CORSA database, represents a valuable resource for ongoing and future OMICS-based CRC projects. CORSA is open for cooperative research projects; expressions of interest are more than welcome.

## Figures and Tables

**Table 1:** Demographic and clinical characteristics of CORSA participants (as of May 2020).

	Colorectal Cancer Study of Austria (*n* = 13,573)			
	CRC	HR ^a^	LR ^b^	Controls ^c^
	1551	2132	3745	6145
Gender *n* (%)				
Male	947 (61.1)	1468 (68.9)	2254 (60.2)	2660 (43.3)
Age *n* (%)				
<50	157 (10.1)	189 (8.9)	485 (13.0)	1738 (28.3)
50–59	259 (16.7)	451 (21.1)	926 (24.7)	1583 (25.8)
60–69	429 (27.7)	741 (34.8)	1253 (33.4)	1546 (25.1)
≥70	706 (45.5)	751 (35.2)	1081 (28.9)	1278 (20.8)
Stage *n* (%) ^d^				
I	423 (27.3)	–	–	–
II	395 (25.5)	–	–	–
III	410 (26.4)	–	–	–
IV	213 (13.7)	–	–	–
Missing	110 (7.1)	–	–	–
Site *n* (%) ^e^				
Colon-distal	404 (26.0)	–	–	–
Colon-proximal	441 (28.5)	–	–	–
Rectum	686 (44.2)	–	–	–
Unknown	20 (1.3)	–	–	–

CORSA participants with multiple recruitment time points were classified according to their most severe histopathological finding. In addition, histopathological findings are recorded at each CORSA participation. ^a^ HR: high-risk adenomas. ^b^ LR: low-risk adenomas (adenomas are classified according to their most severe finding). ^c^ Controls are defined as patients with a negative colonoscopy finding (free of polyps and carcinoma). ^d^ UICC stage based on the TNM Classification of Malignant Tumors. ^e^ Localization of polyps can be provided from clinical records. Distal colon: sigmoid colon, descending colon, splenic flexure. Proximal colon: transverse colon, hepatic flexure, ascending colon, cecum, appendix. Rectum: rectum, rectosigmoid junction.

**Table 2:** CORSA data resources: questionnaire data and clinical databank.

CORSA Questionnaire	
Category	Assessed Variable
Demographics	Name, gender, date of birth
Height	Height in cm
Weight	Weight in kg
BMI	Defined as kg/m^2^
Family status	Single, married, divorced, widowed, life partnership
Education status	Elementary school, secondary school, matriculation, university
Employment	Employed, retired, unemployed, home keeping
Smoking	Current or former smoker (age at start/stop, cigarettes per day)
	Never smoker
Alcohol consumption	Abstainer, former consumer, consumer (self-assessment)
Diet	Self-assessment of food frequency intake
Diabetes	Diabetes status, treatment (dietary restriction, insulin, medication name), year of diagnosis
	HbA1c level
**CORSA Databank: Abstracted Clinical Data**	
**Category**	**Assessed Variable**
Family history of CRC	First- or second-degree relatives
Colonoscopy	Date and site of diagnostic colonoscopy (resident physician, hospital)
Surgery	Date and type of curative surgery
Histological findings	Invasive carcinoma
	Histology, amount, and size of polyps
Mutation status	*KRAS*, *NRAS*, *BRAF*, *PIK3CA*, *MSI*
Inflammatory Bowel Disease (IBD)	Crohn’s disease, ulcerative colitis, e.g., (pathologically confirmed)
Localization	Appendix, caecum, valvula, colon ascendens, flexura coli dextra, colon transversum, flexura coli sinistra, colon descendens, sigmoid colon, rectosigmoid junction, rectum, anus
Grading	Cell differentiation and growth rate, G1–G4
TNM staging (UICC system)	Progression and spread in the body
	Extent (size) of tumor (T): Tis, T0–T4
	Spread to nearby lymph nodes (N): N0–N2
	amount of positive and resected nodes
	Spread (metastasis) to distant sites (M): M0, M1
Cancer stage (UICC system)	Stage 0–IV
Neoadjuvant treatment	Neoadjuvant radiation, chemotherapy, radio-chemotherapy
	Adjuvant radiation, chemotherapy, radio-chemotherapy
	Palliative radiation, chemotherapy, radio-chemotherapy, and amount of administered therapy cycles
Second primary CRC	Synchronous, metachronous CRC
Secondary malignancies	Any carcinoma other than CRC
Local CRC recurrence	Pathologically confirmed CRC recurrence at the anastomosis or nearby the primary tumor
Distant CRC metastasis	Distant recurrence or spread to distant sites (first three sites of metastases assessed)
Survival data	Date of death, date of last contact, CRC-related cause of death

**Table 3:** Available CORSA data derived from GWAS, metabolomics, folate biomarkers, and leukocyte telomere length analyses.

	CORSA			
Available Data	CRC	HR ^a^	LR ^b^	Controls ^c^
GWAS data	1060	689	–	928
Untargeted metabolomics data	88	200	200	400
Targeted metabolomics data	88	200	200	400
Folate-related biomarker status	245	–	–	–
Leukocyte telomere length	384	544	537	546

^a^ HR: high-risk adenomas. ^b^ LR: low-risk adenomas. ^c^ Controls are defined as patients with a negative colonoscopy finding (free of polyps and carcinoma).

## Data Availability

Paper concept proposals of interest for cooperation studies are welcomed. Please send a short proposal to the corresponding author, Andrea Gsur (andrea.gsur@meduniwien.ac.at). The proposal must include a working title, author information, scientific rationale and objectives, study design (study data, primary analysis plan), and material (genomic DNA or plasma) or data requested.

## Supplementary Materials

The following are available online at https://www.mdpi.com/article/10.3390/biology10080722/s1, Table S1: Complete list of CORSA publications (as of June 2021).
