# Supplementary material for: Colorectal Cancer Study of Austria (CORSA): A Population-Based Multicenter Study
Source: Biology (Basel). 2021 Jul 28;10(8):722. doi: 10.3390/biology10080722 (PMC8389216; doi:10.3390/biology10080722)
Supplement: Supplementary file 1 [file biology-10-00722-s001.zip › biology-1290419-supplementary.pdf]

biology

MDPI

Supplementary

Colorectal Cancer Study of Austria (CORSA): A Population-Based Multicenter Study

Andrea Gsur, Andreas Baierl and Stefanie Brezina

Supplementary Table S1. Complete list of CORSA publications (as of June 2021).

Manuscript title	Year	Journal	DOI
Association of IGF1 and IGFBP3 polymorphisms with colorectal polyps and colorectal cancer risk. **[7]**	2010	Cancer Causes & Control	10.1007/s10552-009-9438-4
No association of XRCC1 polymorphisms Arg194Trp and Arg399Gln with colorectal cancer risk. **[17]**	2011	Cancer Epidemiology	10.1016/j.canep.2011.03.005
MNS16A tandem repeats minisatellite of human telomerase gene: a risk factor for colorectal cancer. **[18]**	2011	Carcinogenesis	10.1093/carcin/bgr053
Association of genetic variants of human telomerase with colorectal polyps and colorectal cancer risk. **[19]**	2012	Molecular Carcinogenesis	10.1002/mc.21911
Differential effects of polymorphic alleles of FGF receptor 4 on colon cancer growth and metastasis. **[20]**	2012	Cancer Research	10.1158/0008-5472.can-11-3654
Bayesian and frequentist analysis of an Austrian genome-wide association study of colorectal cancer and advanced adenomas. **[10]**	2017	Oncotarget	10.18632/oncotarget.21697
Leukocyte telomere length throughout the continuum of colorectal carcinogenesis. **[21]**	2018	Oncotarget	10.18632/oncotarget.24431
Functional Polymorphisms in DNA Repair Genes Are Associated with Sporadic Colorectal Cancer Susceptibility and Clinical Outcome. **[22]**	2018	International Journal of Molecular Sciences	10.3390/ijms20010097
The Immunome of Colon Cancer: Functional In Silico Analysis of Antigenic Proteins Deduced from IgG Microarray Profiling. **[23]**	2018	Genomics, Proteomics & Bioinformatics	10.1016/j.gpb.2017.10.002
Discovery of common and rare genetic risk variants for colorectal cancer. **[24]**	2019	Nature Genetics	10.1038/s41588-018-0286-6
Association analyses identify 31 new risk loci for colorectal cancer susceptibility. **[25]**	2019	Nature Communications	10.1038/s41467-019-09775-w
Plasma metabolites associated with colorectal cancer: A discovery-replication strategy. **[11]**	2019	International Journal of Cancer	10.1002/ijc.32146

Genome-wide association analysis of diverticular disease points towards neuromuscular, connective tissue and epithelial pathomechanisms. **[26]**	2019	Gut	10.1136/gutjnl-2018-317619
Novel Common Genetic Susceptibility Loci for Colorectal Cancer. **[27]**	2019	Journal of the National Cancer Institute	10.1093/jnci/djy099
Plasma metabolites associated with colorectal cancer stage: findings from an international consortium. **[12]**	2020	International Journal of Cancer	10.1002/ijc.32666
Modifiable pathways for colorectal cancer: A Mendelian randomisation analysis. **[28]**	2020	The Lancet Gastroenterology & Hepatology	10.1016/S2468-1253(19)30294-8
Distinct Molecular Phenotype of Sporadic Colorectal Cancers Among Young Patients Based on Multi-omics Analysis. **[29]**	2020	Gastroenterology	10.1053/j.gastro.2019.11.012
Cumulative Burden of Colorectal Cancer-Associated Genetic Variants is More Strongly Associated With Early-onset vs Late-onset Cancer. **[30]**	2020	Gastroenterology	10.1053/j.gastro.2019.12.012
Identification of novel loci and new risk variant in known loci for colorectal cancer risk in East Asians. **[31]**	2020	Cancer Epidemiology, Biomarkers & Prevention	10.1158/1055-9965
Circulating Levels of Insulin-like Growth Factor 1 and Insulin-like Growth Factor Binding Protein 3 Associate With Risk of Colorectal Cancer Based on Serologic and Mendelian Randomization Analyses. **[32]**	2020	Gastroenterology	10.1053/j.gastro.2019.12.020
Physical activity and risks of breast and colorectal cancer: A Mendelian Randomization analysis. **[33]**	2020	Nature Communications	10.1038/s41467-020-14389-8
Landscape of somatic single nucleotide variants and indels in colorectal cancer and impact on survival. **[34]**	2020	Nature Communications	10.1038/s41467-020-17386-z
Genome-wide Modeling of Polygenic Risk Score in Colorectal Cancer Risk **[35]**	2020	The American Journal of Human Genetics	10.1016/j.ajhg.2020.07.006.
Circulating B-vitamin biomarkers and B-vitamin supplement use in relation to quality of life in patients with colorectal cancer: results from the FOCUS consortium **[36]**	2020	The American Journal of Clinical Nutrition	10.1093/ajcn/nqaa422
Circulating folate and folic acid concentrations: associations with colorectal cancer recurrence and survival **[37]**	2020	JNCI Cancer Spectrum	10.1093/jncics/pkaa051
Hemochromatosis genotype is not associated with colorectal cancer risk or age of onset **[38]**	2020	Human Genetics and Genomics Advances	10.1016/j.xhgg.2020.100010
Identifying Novel Susceptibility Genes for Colorectal Cancer Risk From a Transcriptome-Wide Association Study of 125,478 Subjects **[39]**	2020	Gastroenterology	10.1053/j.gastro.2020.08.062

A combined proteomics and Mendelian randomization approach to investigate the effects of aspirin-targeted proteins on colorectal cancer **[40]**	2020	Cancer Epidemiol Biomarkers Prev	10.1158/1055-9965.EPI-20-1176
Multi-omics analysis reveals adipose-tumor crosstalk in colorectal cancer patients **[41]**	2020	Cancer Prevention Research	10.1158/1940-6207
Adiposity, metabolites, and colorectal cancer risk: Mendelian randomization study **[42]**	2020	BMC Medicine	10.1186/s12916-020-01855-9
Genetically predicted circulating concentrations of micronutrients and risk of colorectal cancer among individuals of European descent: a Mendelian randomization study **[43]**	2021	The American Journal of Clinical Nutrition	10.1093/ajcn/nqab003
Genetic architectures of proximal and distal colorectal cancer are partly distinct **[44]**	2021	Gut	10.1101/2020.05.01.20087957
Diet quality indices and dietary patterns are associated with plasma metabolites in colorectal cancer patients **[45]**	2021	European Journal of Nutrition	10.1007/s00394-021-02488-1
Untargeted metabolomics reveals major differences in the plasma metabolome between colorectal cancer and colorectal adenomas **[46]**	2021	Metabolites	10.3390/metabo11020119
Targeted plasma metabolic profiles and risk of recurrence in stage II and III colorectal cancer patients: Results from an international cohort consortium **[47]**	2021	Metabolites	10.3390/metabo11030129
Polymorphisms within autophagy-related genes influence the risk of developing colorectal cancer: a meta-analysis of four large cohorts **[48]**	2021	Cancer	10.3390/cancers13061258
Genome-wide analysis of 944,133 individuals provides insights into the etiology of hemorrhoidal disease **[49]**	2021	Gut	10.1136/gutjnl-2020-323868
Lack of an association between circulating bilirubin levels and gallstone disease with risk of colorectal cancer: A Mendelian randomisation analysis **[50]**	2021	British Journal of Cancer	10.1038/s41416-020-01211-x
Circulating tryptophan metabolites and risk of colon cancer: results from case-control and prospective cohort studies **[51]**	2021	International Journal of Cancer	In press
